# Supplementary figures and images for: Founder effects drive the genetic structure of passively dispersed aquatic invertebrates
Source: PeerJ. 2018 Dec 11;6:e6094. doi: 10.7717/peerj.6094 (PMC6294052; doi:10.7717/peerj.6094)

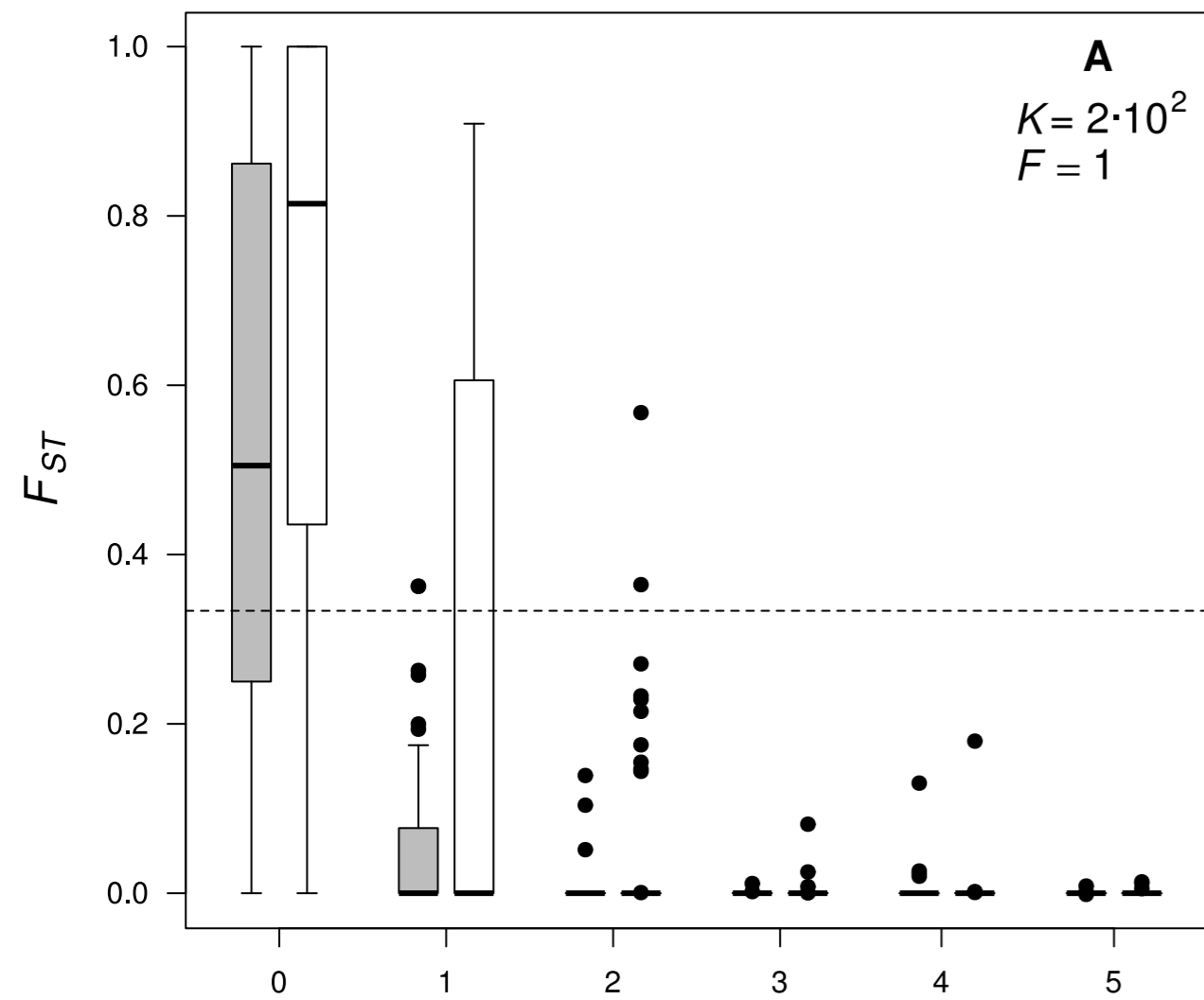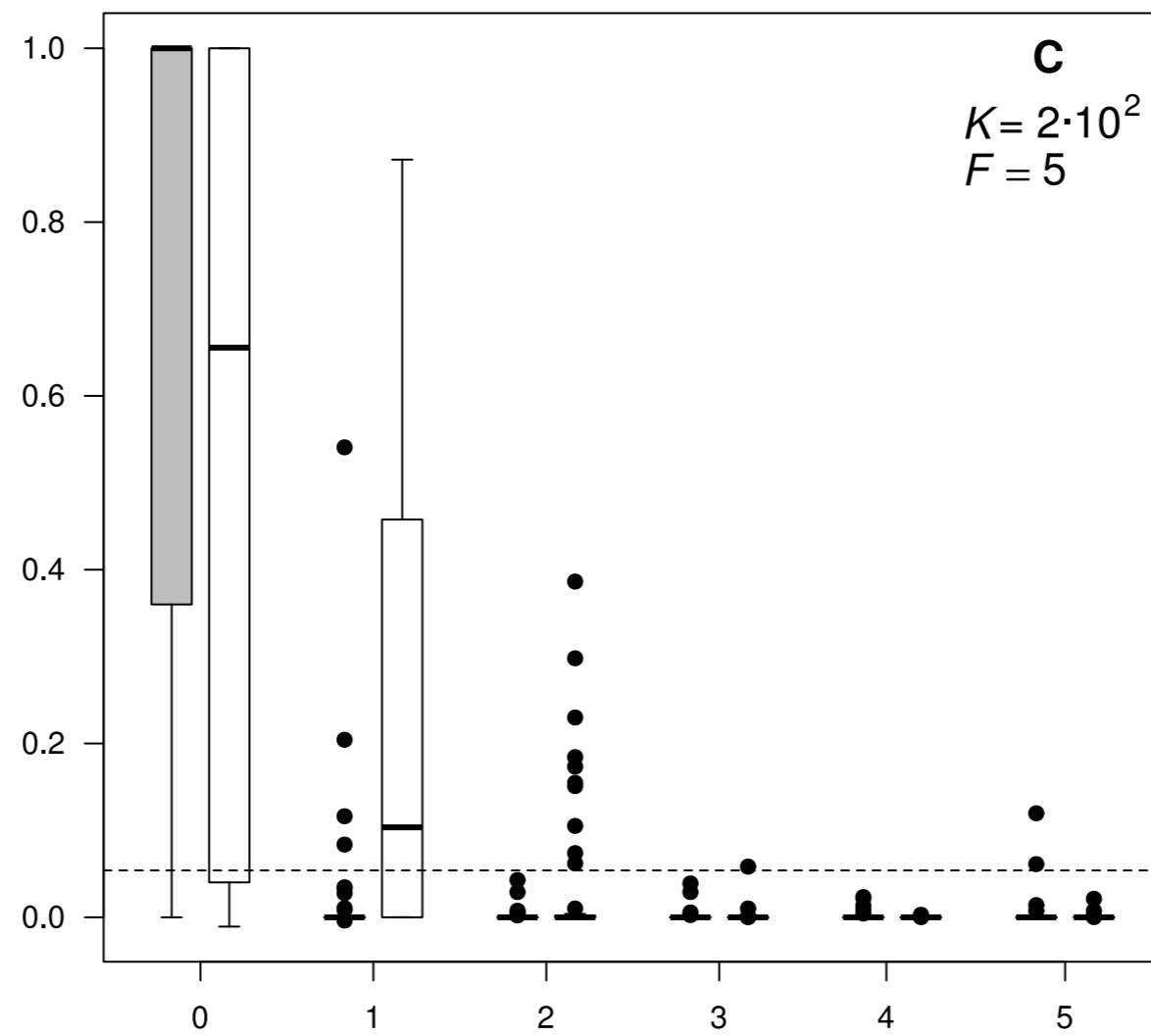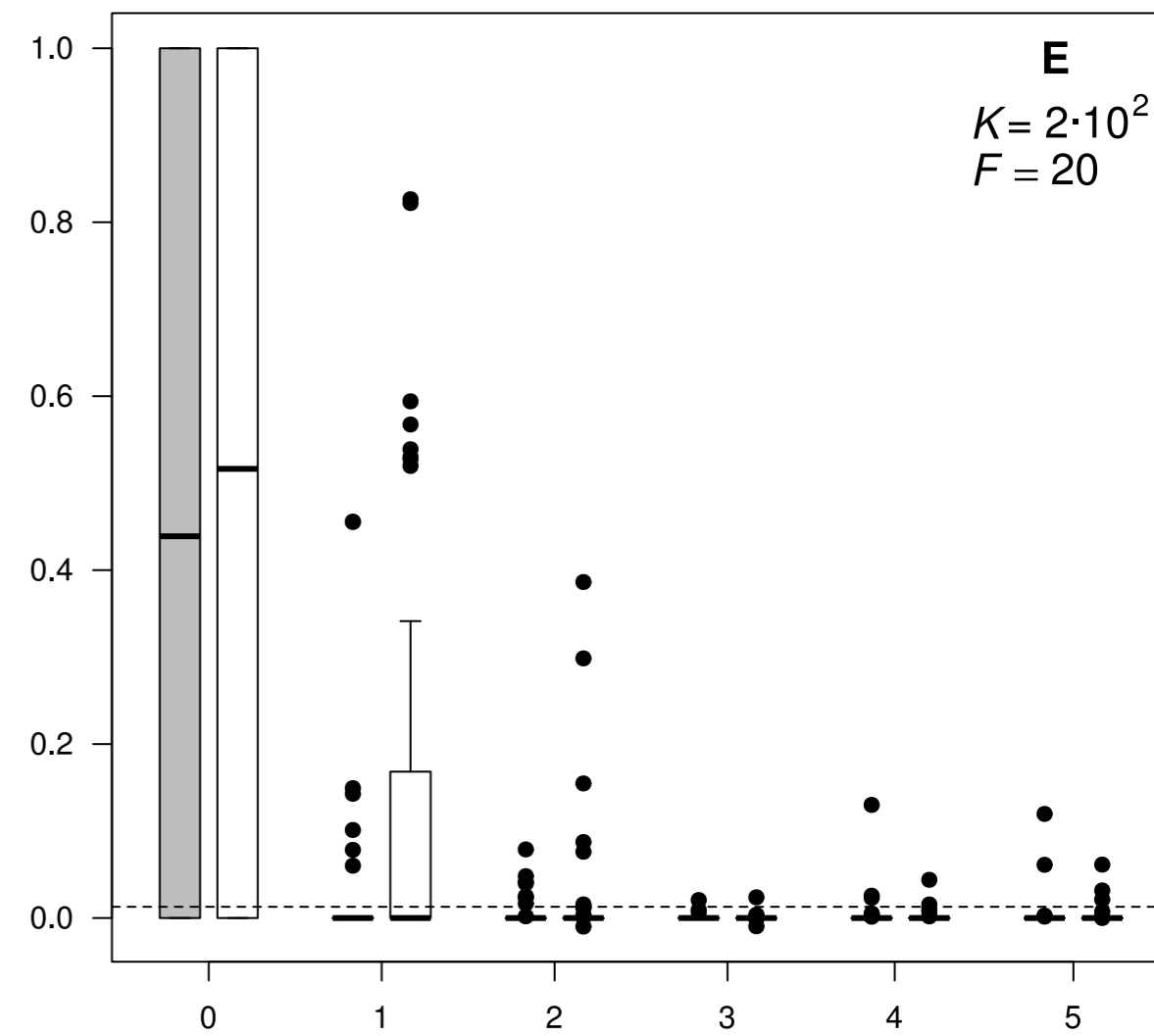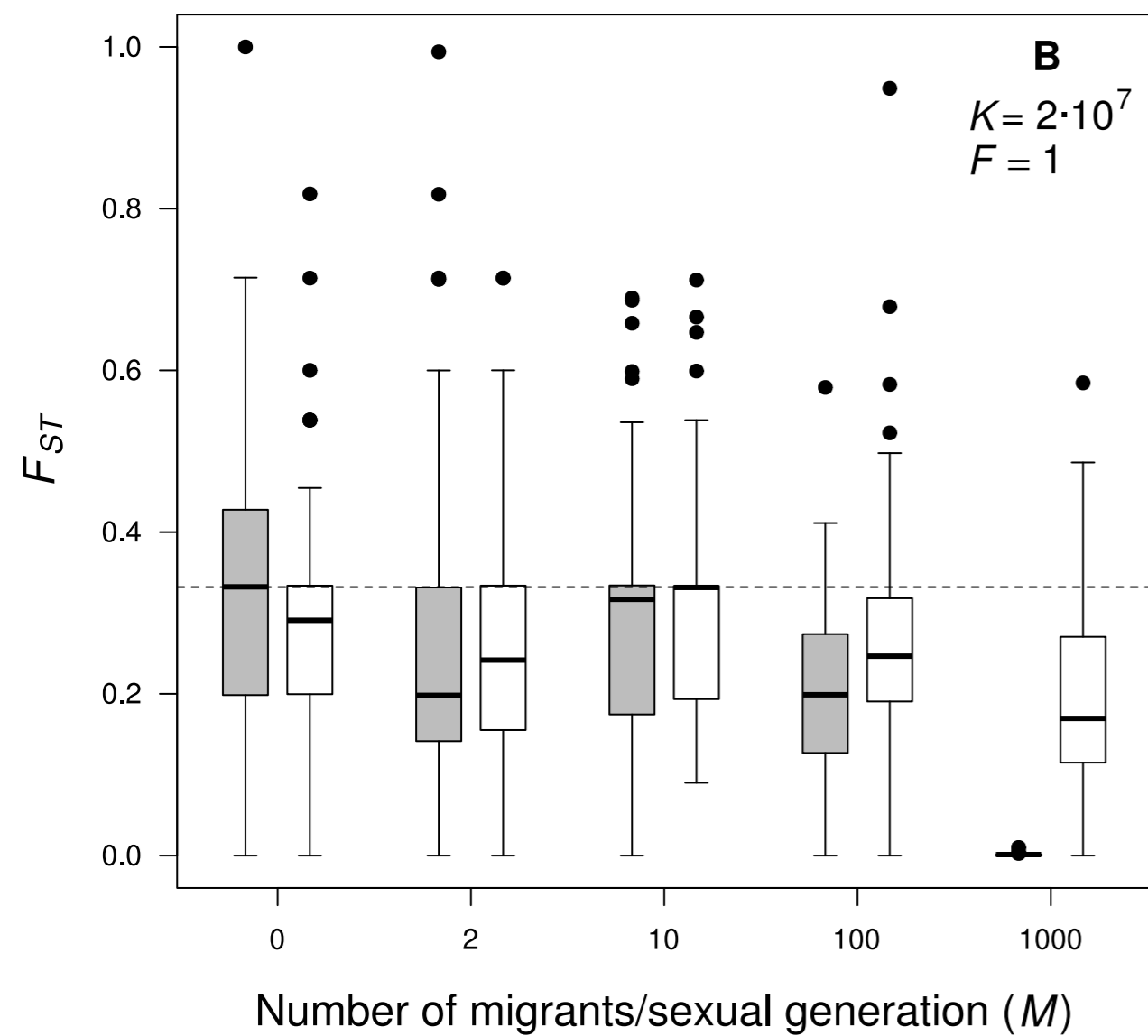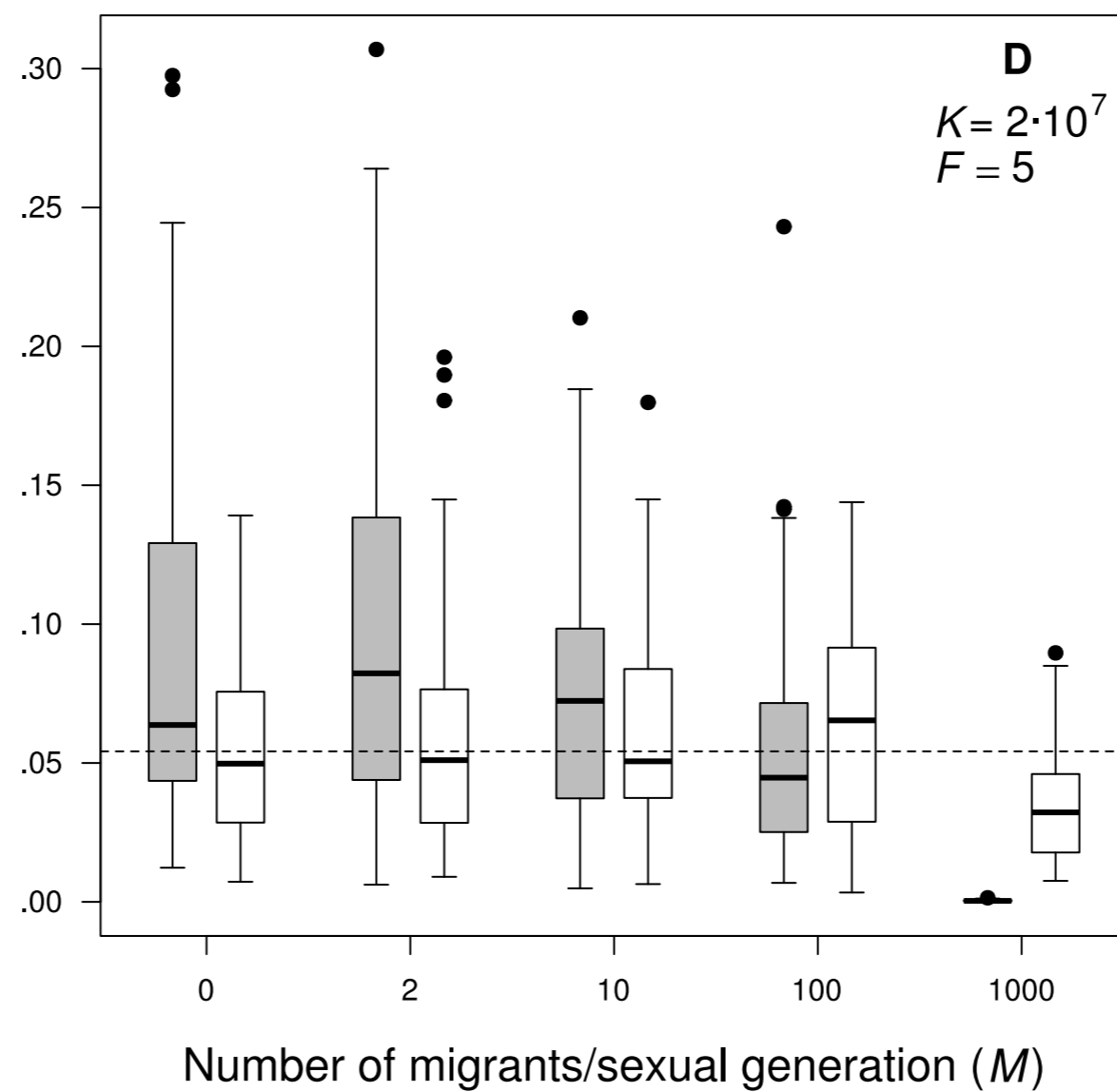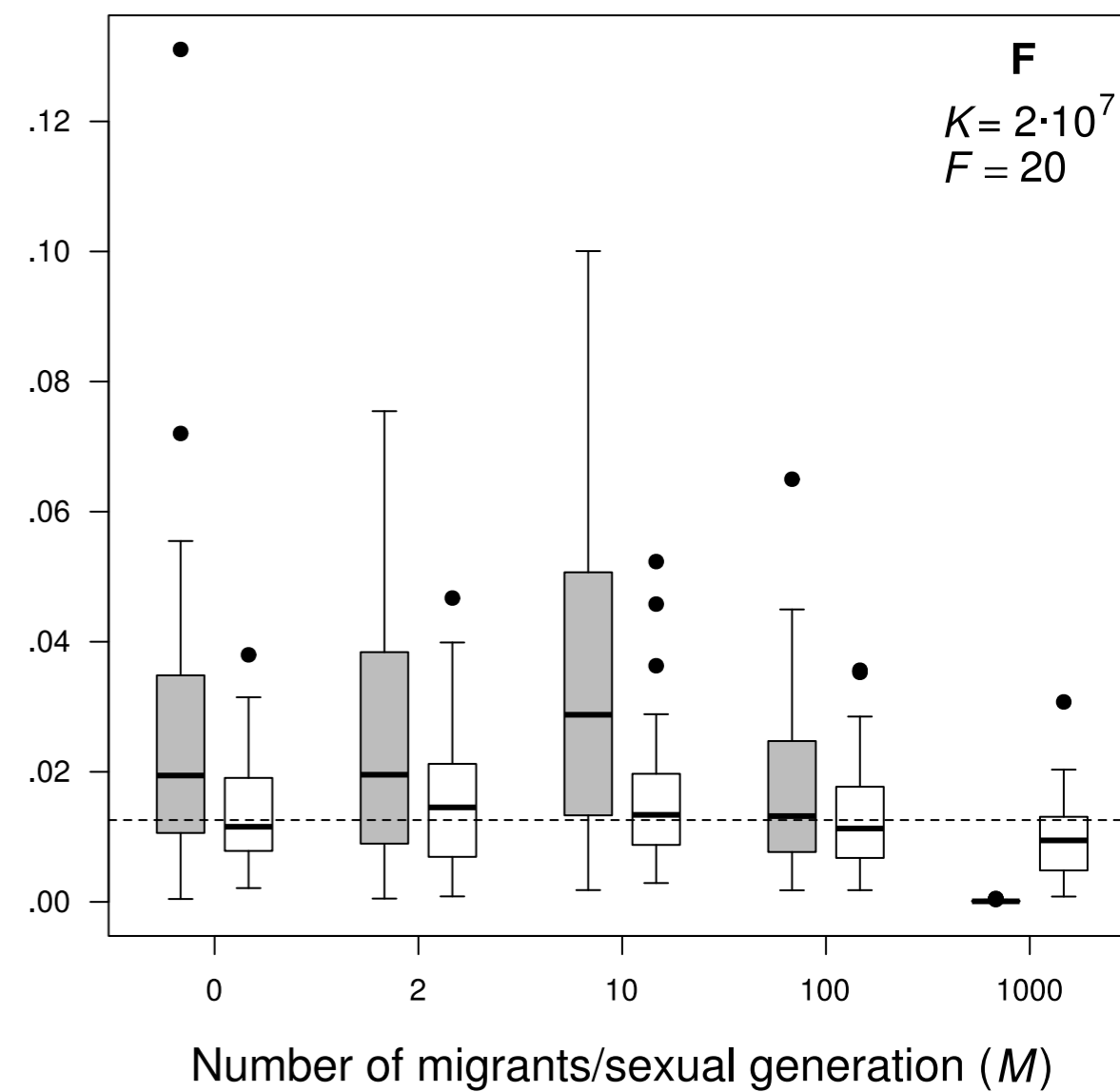

Supplement: Supplemental Information 1 — Population differentiation (FST) after 1,000 sexual generations plotted against migration (M) with and without a diapausing egg bank for (A) K = 2 × 10^2^ and F = 1, (B) K = 2 × 10^7^ and F = 1, (C) K = 2 × 10^2^ and F = 5, (D) K = 2 × 10^7^ and F = 5, (E) K = 2 × 10^2^ and F = 20 and (F) K = 2 × 10^7^ and F = 20. The rest of parameters were r = 0.3d − 1, n = 5 and s = 0. Box plots are based on 50 replicate simulations. Boxes represent 25th/75th percentile and black dots the 5th/95th percentile. Thin black lines and thick gray lines in each bar represent the median and the mean, respectively. Dashed horizontal lines show the initial value of FST after foundation. [file peerj-06-6094-s001.pdf]
