# Supplementary material for: Founder effects drive the genetic structure of passively dispersed aquatic invertebrates
Source: PeerJ. 2018 Dec 11;6:e6094. doi: 10.7717/peerj.6094 (PMC6294052; doi:10.7717/peerj.6094)
Supplement: Figure S1 — Box plot graph of FST and FSTQ values after 1,000 sexual generations with different recombination rates for two different values of fitness components (δ = 10−4 and 10−2d−1) and without a diapausing egg bank. For each of the fitness scenario, the left panel refers to K = 2 × 10^4^ and the right panel, to K = 2 × 10^7^. The rest of parameters were r = 0.3d−1, n = 5, s = 5, F = 1 and M = 2. Data is based on 100 replicates. Boxes represent 25th /75th percentile and black dots the 5th/95th percentile. Thin black lines and thick gray lines in each bar represent the median and the mean respectively. Dashed lines show the initial value of FST after foundation. Asterisks indicate FST statistically different from those without selection (δ = 0) (**, α = 0.05). [file peerj-06-6094-s002.pdf]

Figure legends

Figure S1.- Box plot graph of F_{ST} and F_{STQ} values after 1000 sexual generations with different recombination rates for two different values of fitness components (δ = 10^{-4} and 10^{-2} d^{-1}) and without a diapausing egg bank. For each of the fitness scenario, the left panel refers to K = $2{\cdot}10^4$ and the right panel, to K = $2{\cdot}10^7$. The rest of parameters were r = 0.3 d^{-1}, n = 5, s = 5, F =1 and M = 2. Data is based on 100 replicates. Boxes represent 25th /75th percentile and black dots the 5th/95th percentile. Thin black lines and thick gray lines in each bar represent the median and the mean respectively. Dashed lines show the initial value of F_{ST} after foundation. Asterisks indicate F_{ST} statistically different from those without selection (δ = 0) (**, α = 0.05).

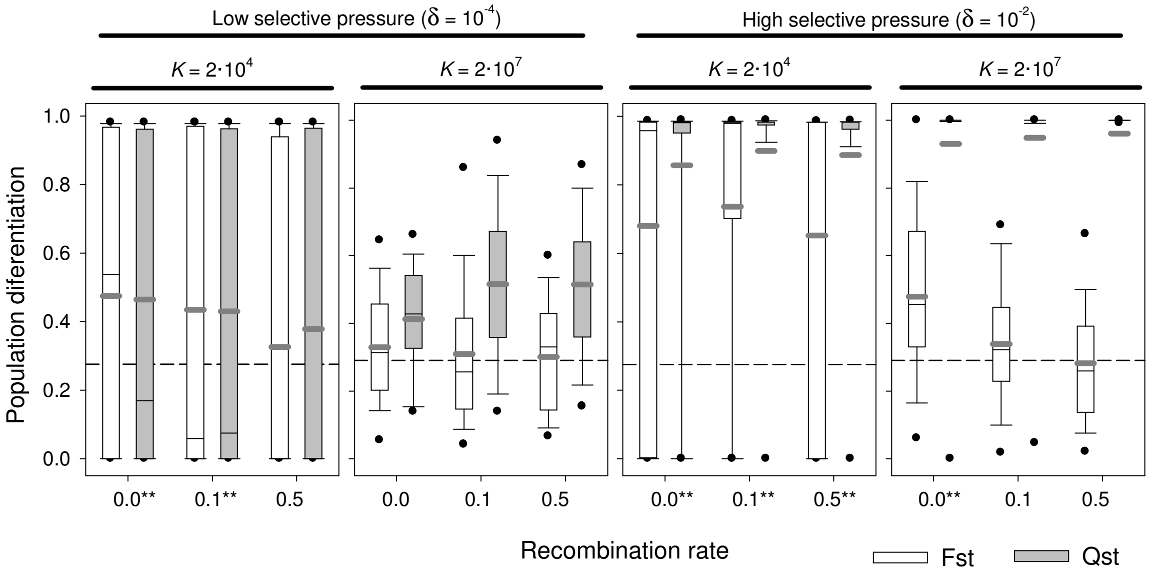

Figure S1.
